# Strategy for Calculating Magnesium Sulfate Dose in Obese Patients: A Randomized Blinded Trial

**DOI:** 10.1155/2022/8424670

**Published:** 2022-11-08

**Authors:** Sebastião E. Silva Filho, Omar S. Klinsky, Miguel A. M. C. Gonzalez, Sandro Dainez, Flavio Angelis, Joaquim E. Vieira

**Affiliations:** ^1^Hospital da Beneficência Portuguesa de Santos, Santos, Brazil; ^2^Department of Surgery, Faculdade de Medicina da Universidade de São Paulo, São Paulo, Brazil

## Abstract

**Background:**

Magnesium sulfate has analgesic properties during the postoperative period. However, there is a knowledge gap in pharmacology related to the use of the real, ideal, or corrected ideal body weight to calculate its dose in obese patients. This trial compared postoperative analgesia using actual and corrected ideal body weight.

**Methods:**

Seventy-five obese patients scheduled to undergo laparoscopic gastroplasty or cholecystectomy under general anesthesia were randomly assigned to three groups. The patients in the control group did not receive magnesium sulfate; the other two groups received magnesium sulfate at 40 mg·kg^−1^ of actual body weight or corrected ideal body weight.

**Results:**

In patients with body mass index >30 mg·kg^−2^ (mean body mass index ranging from 32.964 kg·m^−2^ to 33.985 kg·m^−2^, according to the groups) scheduled for video laparoscopic cholecystectomy, there were no differences in the blood magnesium concentrations in the groups receiving magnesium sulfate throughout the study, regardless of whether the strategy to calculate its dose was based on total or corrected ideal body weight. Patients in the groups receiving magnesium sulfate showed a significant reduction in morphine consumption (*p* ≤ 0.001) and pain scores (*p*=0.006) in the postoperative period compared to those in the control group. There were no significant differences in morphine consumption (*p*=0.323) or pain scores (*p*=0.082) between the two groups receiving magnesium sulfate. There were no differences in the total duration of neuromuscular block induced by cisatracurium among the three groups (*p*=0.181).

**Conclusions:**

Magnesium sulfate decreased postoperative pain and morphine consumption without affecting the recovery time of cisatracurium in obese patients undergoing laparoscopic cholecystectomy. Strategies to calculate the dose based on the actual or corrected ideal body weight had similar outcomes related to analgesia and the resulting blood magnesium concentration. However, as the sample in this trial presented body mass indices ranging from 30.11 kg·m^−2^ to 47.11 kg/m^−2^, further studies are needed to confirm these findings in more obese patients, easily found in centers specialized.

## 1. Introduction

In addition to its usefulness in various fields of medicine [1–8], magnesium sulfate (MS) is a good adjunct analgesic [9] because it blocks calcium channels and N-methyl-d-aspartate (NMDA) receptors [4, 5]. It has been administered as a bolus [10, 11] or in combination with continuous infusion [12, 13] without a clear advantage over others.

Worldwide, the increase in obesity prevalence [14] is associated with an increase in the frequency of obese patients in surgery rooms. Obese patients also receive MS in many situations [15]. It is necessary to adjust the dosage of some drugs in obese individuals because of pharmacokinetic changes caused by increased fat tissue [16, 17]. However, to our knowledge, no study has analyzed the best way to calculate the MS dose in obese patients using actual, ideal, or corrected ideal body weight.

This trial compared the analgesic effects of MS in obese patients using two strategies to calculate the dose (real body weight and corrected ideal body weight). The primary objective was to compare the blood magnesium concentrations in both groups. The secondary objectives were to compare analgesia and the time frame to the recovery of 90% of the train of four (TOF) after cisatracurium administration. We recorded the blood magnesium concentration at planned moments, morphine consumption, pain scores during the postoperative period of 24 h, and total duration of onset and total neuromuscular blockade [18].

## 2. Materials and Methods

This is a randomized controlled trial with blinding of patients and clinical staff, carried out at the hospital of the Sociedade de Beneficência Portuguesa de Santos, SP. Data were collected from August 26, 2019, to November 12, 2020. This manuscript adheres to the Consolidated Standards of Reporting Trials (CONSORT) guidelines. This study was approved by the Institutional Review Board (IRB) of the Universidade de Taubaté, SP, Brazil (IRB number 09006119.2.0000.5501). Written informed consent was obtained from all participants prior to their participation in the study. The trial was registered at clinicaltrials.gov (NCT04003688; principal investigator: Sebastião Ernesto da Silva Filho; registration date: June 24, 2019) before patient enrollment.

### 2.1. Study Population

The inclusion criteria were as follows: patients aged 18–60 years, with American Society of Anesthesiologists physical status II and body mass index (BMI) >30 kg·m^−2^, scheduled for video laparoscopic cholecystectomy. The exclusion criteria were as follows: history of allergy to any component of the study protocol, refusal to participate or sign the informed consent form, neuromuscular disorders, cardiac conduction block other than first-degree atrioventricular block, illicit drug use, psychiatric disorders that compromise the assessment of symptoms, use of calcium channel blockers, and renal failure.

The sample was based on a trial by Kizilcik and Koner [15]; they administered MS 30 mg·kg^−1^ in obese patients with less pain than the control group (9.50 ± 2.98 vs. 12.65 ± 2.34) 60 min postoperatively. The mean difference between the actual and corrected ideal body weights of the obese population in our hospital over the last 3 months was approximately 20%. To our knowledge, no study has compared the actual and corrected ideal body weights to calculate the MS dose in obese patients. Therefore, we used this 20% difference as a surrogate for the difference between the means and estimated; for a confidence level of 95% and a power of 80%, a sample of 19.43 participants [19] per group was needed. We increased the number to 25 to compensate for possible losses.

Among the patients who agreed to participate in the study, 75 individuals were selected and divided electronically into three groups using https://www.random.org/, which provides truly random numbers originating from atmospheric noise. In the control group (CG), 25 patients received general anesthesia (GA) only. In the real body weight group (RWG), the patients received GA and MS at a dose of 40 mg·kg^−1^ of their real body weight. In the corrected ideal body weight group (CWG), the patients received GA and MS at a dose of 40 mg·kg^−1^ of their corrected ideal body weight, calculated using the Broca's [20] index: male = height − 100 + {0.4 × [actual − (height − 100)]} and female = height − 105 + {0.4 × [actual − (height − 105)]}. The weights of all patients were measured during preanesthetic consultation using a calibrated electronic scale.

The electronic drawing allowed 75 envelopes with information about the related groups and procedures performed by a professional blinded to the study protocol. Another team member, not involved in any other task of this trial, prepared the concealed solutions.

### 2.2. Anesthetic Technique

The participants were monitored with continuous electrocardiography, pulse oximetry, noninvasive blood pressure on a multiparameter monitor (Mindray, model IPM-9800, China), and hypnosis level (patient state index, SedLine® Sedation Monitor, USA) before receiving any medication. The patients were also connected to a neuromuscular function (NMF) monitor (TOF-Watch SX; Ireland).

The study included a group of 10 patients with a BMI of 20–30 kg·m^2^ who received 40 mg·kg^−1^ MS and underwent the same protocol as participants in the RWG. This group, called the nonobese group (NOG), provided the standard magnesium concentration generated after MS administration in nonobese patients, and we could see how different the outcomes would be in each group of obese participants. The participants in this group followed the same inclusion and exclusion criteria as the obese patients in the study, except for their weight.

To prevent nausea and vomiting and reduce postoperative pain, patients were also administered dipyrone 15 mg·kg^−1^, clonidine 2 *μ*g·kg^−1^, dexamethasone 4 mg, ketoprofen 100 mg, ondansetron 4 mg, and lidocaine 1.5 mg·kg^−1^ just before anesthetic induction. Simultaneously, the patients received the concealed solution, followed by preoxygenation with a fraction of inspired oxygen = 1 for 3 min, and propofol in target-controlled infusion (TCI) to reach a concentration of 4 *μ*g·mL^−1^, guided by a hypnosis monitor. After appropriate hypnosis (Patient State Index (PSI) < 50), calibration of the neuromuscular function monitor was performed using TOF monitoring, followed by 0.15 mg·kg^−1^ cisatracurium intravenously and remifentanil infusion through TCI until 5 ng·mL^−1^ effect target concentration was reached. Anesthesia was maintained with propofol in TCI to maintain a PSI of 25–50, remifentanil in TCI (target of 3 to 5 ng·mL^−1^, according to cardiac rate and blood pressure), and cisatracurium 0.03 mg·kg^−1^ if TOF > 2 count. Administration of cisatracurium was avoided during the last 20 min of surgery. At the end of the surgery, patients with TOF > 2 were administered 20 *μ*g·kg^−1^ atropine and 40 *μ*g·kg^−1^ neostigmine.

Before extubation, all patients were administered morphine (0.05 mg·kg^−1^) and dipyrone (15 mg·kg^−1^). Five min after anesthetic recovery and every 30 min thereafter, patients received another dose of morphine if the pain score was greater than 3 points on the verbal numeric scale (VNS: 0 (no pain) to 10 (the highest possible pain) points). In the ward, they received 1 g of dipyrone intravenously (every 6 h), 10 mg of nalbuphine hydrochloride (every 8 h), and 0.05 mg·kg^−1^ of morphine if their pain score was greater than 3 points in the VNS. All patients were discharged from the hospital the morning after the surgery.

### 2.3. Outcomes Measured

Immediately after venipuncture, the first blood sample (2 mL) was collected to measure blood magnesium concentration. The concealed solution was then infused for 10 min. The concealed solution consisted of 100 mL of saline solution or saline solution with MS, for a total of 100 mL, depending on the group.

Blood samples were collected to measure the blood magnesium concentration in the arm contralateral to the arm receiving the medication while maintaining an indwelling catheter. The collection times were as follows: venipuncture (before any medication) and 15, 30, 60, 120, and 240 min. Magnesium concentrations were measured in the hospital laboratory using mg·dL^−1^ as the unit of measurement. Patients and everyone involved in the research were blinded to Mg levels.

The analgesic effect of different doses of MS was assessed using the following outcomes: VNS at 5 min after extubation; at 30, 60, 120, and 240 min; the highest pain score in the perioperative period during hospital stay (VNS); and morphine consumption during the hospital stay. Morphine was administered on demand or when the pain score was greater than 3 points (VNS).

The effects of MS on cisatracurium pharmacology were evaluated using the onset time (TOF = 0) and total duration (time to TOF T4/T1 = 90%). Differences in blood magnesium concentrations between groups were compared using the concentrations verified at the collection times defined in the study protocol. The NOG (BMI of 20–30 kg·m^2^) was used as a reference to show how close or far the average magnesium concentration in obese patients receiving different doses of MS was from the average concentration in nonobese patients at those times.

### 2.4. Statistical Analysis

The hypotheses of interest were tested using parametric analysis of variance (ANOVA) or repeated-measures ANOVA when observations were taken over time. Normality was verified using the Shapiro–Wilk test, and samples without a normal distribution were compared using the Kruskal–Wallis test. Results with a descriptive level of less than 5% (*p* < 0.05) were considered significant. When comparisons between groups showed a difference, we applied the Bonferroni post hoc test.

Blood magnesium concentrations during the perioperative period were tested using two-way ANOVA for repeated measurements. Violation of sphericity was demonstrated using Mauchly's test. The Greenhouse–Geisser method was used to correct this bias.

## 3. Results

**Figure 1:**
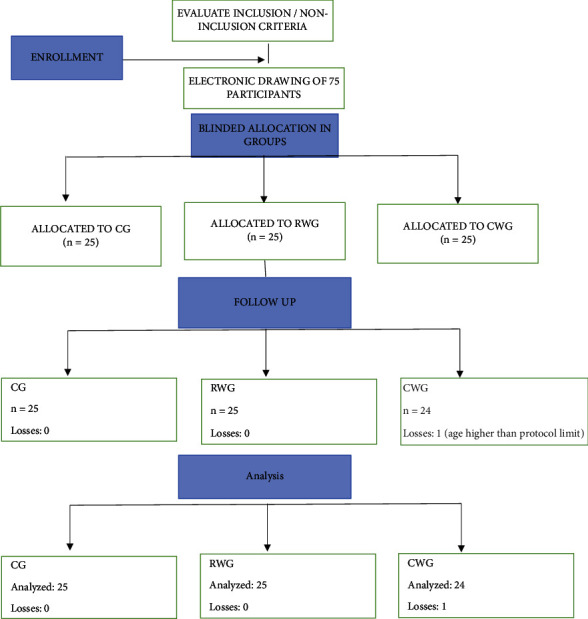
CONSORT Flowchart. CG, control group; RWG, real body weight group; CWG, corrected ideal body weight group.

One patient was excluded from the study (CONSORT flowchart; Figure 1).  Table 1 shows similar durations of anesthesia, weight, height, and BMI.

**Figure 2:**
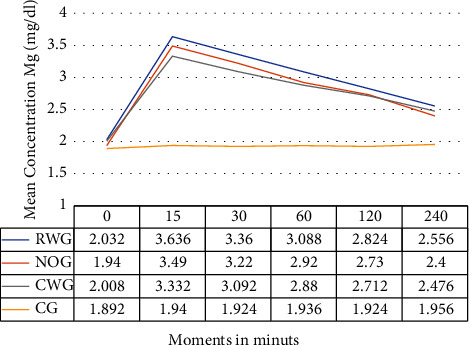
Blood magnesium: comparison of mean concentration throughout the study. RWG, real body weight group; NOG, nonobese group; CWG, corrected ideal body weight group; CG, control group.

As expected, the analysis showed similar blood magnesium concentrations in all measurements performed in the CG (Figure 2). RWG and CWG showed concentrations similar to those of CG at time zero. However, they showed an increase in the Mg concentration at 15 min, with a progressive decay in the subsequent moments. There were no significant differences in the blood magnesium concentrations between the RWG and CWG at the collection times provided by the study protocol. The blood magnesium concentration in the MS-treated obese group was similar to that in the NOG (Table 2 and Figure 2).

In the CWG, the participants' average real body weight (92.54 kg) was compared to their average corrected ideal body weight (73.54 kg), with a mean difference of 18.72 kg. Therefore, these participants received a 21.6% lower MS dose than they would have received if the dose had been calculated based on their actual weight.

None of the patients reported any pain upon awakening. Data on the highest postoperative pain scores and morphine consumption during their hospital stay were not normally distributed (Shapiro–Wilk test). The pain scores at 30, 60, 120, and 240 min were normally distributed.

Regarding the highest postoperative pain scores during their hospital stay, the comparison between the groups showed a significant difference (*p*=0.006, Kruskal–Wallis test). The post hoc test for multiple comparisons showed a statistical difference between the RWG and CG (*p*=0.005, Bonferroni) and between the CWG and CG (*p*=0.016, Bonferroni), but there were no statistical differences between the RWG and CWG (*p*=0.082, Bonferroni; Table 3).

Participants in the CWG had lower pain scores than those in the CG (*p* < 0.05) at 30 min (one-way ANOVA; Bonferroni). Participants in the RWG showed lower pain scores than those in the CG (*p* < 0.05) at 30 and 60 min (one-way ANOVA; Bonferroni correction). There were no statistical differences between the groups at any other time point (Table 4).

There were differences in morphine consumption during the hospital stay between the groups (*p* ≤ 0.001, Kruskal–Wallis test). Multiple post hoc comparisons adjusted by Bonferroni correction showed a similarity in morphine consumption between the RWG and CWG (*p*=0.108; corrected *p*=0.323). The RWG (*p* ≤ 0.001; corrected *p* ≤ 0.001) and CWG (*p*=0.013; corrected *p*=0.040) had significantly lower morphine consumption than CG (Table 3).

The latency (*p*=0.651) and total duration of action (*p*=0.181) of cisatracurium were independently analyzed using a one-way ANOVA (Welch's correction due to unequal variance). No statistical differences existed between the groups in either of the two variables studied (Table 5).

## 4. Discussion

In this randomized trial, administering a bolus dose of MS increased serum magnesium levels and improved postoperative analgesia in obese patients with an average BMI of 33 kg·m^−2^, undergoing laparoscopic cholecystectomy. Blood magnesium concentration, postoperative analgesia, and recovery from neuromuscular blockade after MS administration were similar in this population and compared to 10 controls with normal body mass index regardless of whether the dose was calculated using actual or corrected ideal body weight.

An increase in adipose tissue and muscle mass modifies the pharmacokinetics of many drugs [16, 17, 21, 22]. Furthermore, obesity-associated diseases reduce the physiological reserves in this population [23].

Despite the benefits of MS in various areas of medicine [1–8], it has side effects, such as the delayed recovery of neuromuscular function and orotracheal extubation [24]. For this reason, establishing the ideal dosing paradigm for MS in obese patients to maximize analgesia without resulting in unsafe serum levels or undesirable prolongation of neuromuscular blockade is important.

In this trial, there was a 22% difference between the actual body weight and the corrected ideal body weight in the CWG. However, investigators were surprised to note that the 22% difference in MS dosing did not result in differences in the Mg concentration between the CWG and RWG groups, as shown in Table 2 and Figure 2. Even after receiving MS doses with a 21.6% difference, the MS groups had similar blood magnesium concentrations (Table 2 and Figure 2). These concentrations were similar to the blood magnesium concentrations in the NOG (10 patients). These concentrations were always within the safe values for study patients [25], as reported by Taheri et al. [10]. Although they did not analyze the analgesic properties, Brookfield et al. [26] found that obese pregnant women needed a longer time to reach a therapeutic concentration of magnesium in the blood for seizure prophylaxis in preeclampsia, which was higher than the average concentration in the present trial and may be higher than the level needed to achieve postsurgical analgesia, which was higher than the average concentration in the present trial.

One gram of MS contains 98.6 mg of magnesium (Fresenius Kabi Canada, Toronto, ON). According to the mean actual body weight in the RWG (94.32 kg) and corrected ideal body weight in the CWG (73.54 kg), the participants received 3,772.8 and 2,941.6 mg of magnesium, respectively. Based on the calculated total blood volume of 70 mL·kg^−1^ of ideal body weight [27–29], we estimated an average plasma volume of 2700 mL in both groups. Therefore, participants in the CWG and RWG received 290 and 372 mg of Mg, respectively. Subsequently, ignoring the tissue distribution volume, there was an increase in blood magnesium concentration of 1.07 and 1.38 mg·mL^−1^, respectively. Similar concentrations were reached 30 min after administration, and the highest peak was reached in the first 15 min.

The average body content of magnesium is 24 g in individuals weighing 70 kg [30, 31]. Only approximately 0.3% of this content is distributed in the plasma [30, 31]. This is a possible cause of the rapid balance in concentration and similar analgesia between the groups that received MS. The patients had an average increase in body Mg of 1.2% in the CWG and 1.55% in the RWG. Pascoal et al. [32] compared two groups of 31 patients who underwent MS treatment to prevent preeclampsia. After an initial MS dose of 6 g, patients received a continuous infusion of 1 or 2 g·h^−1^. The initial concentration was statistically equal between the groups (3.7 mEq·L^−1^; *p*=0.96). Subsequently, the concentrations increased in the group that received an infusion of 2 g·h^−1^ and were relatively static in the group that received 1 g·h^−1^. The authors concluded that an infusion of 1 g·h^−1^ could be as effective as an infusion of 2 g·h^−1^, with a small reduction in side effects. This knowledge may be applied to the use of MS for analgesic purposes.

In this trial, patients who received MS had lower mean postoperative pain scores and lower morphine consumption. These results, which have been reported in other studies [9–13], are attributed to the action of magnesium on calcium channels and NMDA receptors [4, 5]. We compared pain at planned moments (30, 60, 120, and 240 min; Table 4) when patients were asked about pain. At 30 min, both groups that received MS experienced less pain than the CG. At 60 min, the RWG experienced less pain. After 30 min, there were no differences in the pain scores among the three groups. When asked about the highest pain during hospitalization, participants in the RWG and CWG were similar, although the CWG received 21.6% less MS, and both groups showed similar morphine consumption rates. However, both patients experienced less pain and morphine consumption than patients in the CG. Maintaining an infusion of MS can produce prolonged analgesia.

MS did not alter the onset or duration of neuromuscular blockade induced by cisatracurium. Germano et al. [33] did not find any difference in the latency of rocuronium 0.6 mg·kg^−1^ after MS. Czarnetzki et al. [34] found a significant reduction in latency (average of 77 vs. 120 s) of rocuronium 0.6 mg·kg^−1^ after MS at higher doses than in the study by Germano et al. (60 mg·kg^−1^). The difference in the time gap between the administration of MS and rocuronium might have interfered with these results. Czarnetzki [35] found a longer mean total recovery time after MS 60 mg·kg^−1^ (73.2 (SD = 22) vs. 57.8 (SD = 14.2) min in the CG). The absolute onset times in the present study cannot be compared to those reported by Germano et al. [33] and Czarnetzki et al. [34] because they used different neuromuscular blockers. Czarnetzki et al. [35] used higher MS doses, excluding the comparison with the present trial.

Sugimoro et al. [36] reported a reduction in the production of inflammatory cytokines (tumor necrosis factor and interleukin 6) in pregnant women who received MS. This mechanism should be investigated in the context of substance use for analgesic purposes.

The strengths of this study include methodological caution and the fear of administering MS to an underexplored population regarding the analgesic and unwanted effects of this drug. Thus, it is safe to calculate the MS dose using total body weight, although this is not always necessary.

The limitations of this study include the relatively low BMI range of participants. The average BMI was 35 kg·m^−2^ (32.964 kg·m^−2^ to 36.478 kg·m^−2^). This is less than what is seen in several specialized centers that easily treat patients weighing >40−50 kg·m^−2^. Although we observed a safe use of MS based on actual body weight, which increased the magnesium blood concentration in proportion to the increasing dose, more research is necessary for patients with a higher BMI. In addition, a larger sample size may bring in more normalized variables and give the test more power.

In patients with higher body mass indexes, dosing based on total body weight could result in toxic magnesium levels and should not be performed outside the protection of close monitoring and rigorous research protocol.

## 5. Conclusion

Magnesium sulfate decreased postoperative pain and morphine consumption without affecting the recovery time of cisatracurium in obese patients undergoing laparoscopic cholecystectomy. Strategies to calculate the dose based on the actual or corrected ideal body weight had similar outcomes related to analgesia and the resulting blood magnesium concentration. However, as the sample in this trial presented body mass indices ranging from 30.11 kg.m-2 to 47.11 kg/m-2, further studies are needed to confirm these findings in more obese patients, easily found in centers specialized.

## Figures and Tables

**Table 1:** Demographic data and duration of anesthesia.

		Duration (min)	Age (years)	Weight (kg)	Height (m)	BMI (kg·m^−2^)
CG (*n* = 25)	*N*	25	25	25	25	25
	Mean	55.960	43.720	95.160	1.648	34.969
	CI 95%^*∗*^	53.353	39.523	90.589	1.615	33.985
		58.567	47.917	99.731	1.681	35.952
CWG (*n* = 24)	*N*	24	24	24	24	24
	Mean	58.125	42.125	92.542	1.642	34.285
	CI 95%^*∗*^	54.671	37.773	87.906	1.610	32.964
		61.579	46.477	97.177	1.674	35.606
RWG (*n* = 25)	*N*	25	25	25	25	25
	Mean	57.000	41.760	94.320	1.644	34.801
	CI 95%^*∗*^	53.861	37.630	88.167	1.616	33.124
		60.139	45.890	100.473	1.672	36.478
*p* value		0.594	0.770	0.757	0.960	0.749

CG, Control group; CWG, corrected ideal weight group; RWG, real weight group; BMI, body mass index; CI, confidence interval.

**Table 2:** Mean and standard deviation of blood magnesium concentration in three groups over time (mg dL^−1^).

Time	Group	*n*	Mean	Standard deviation	*p* value
T0	NOG	10	1.94	0.39	0.753
	CWG	24	2.01	0.30	
	RWG	25	2.03	0.32	

T15	NOG	10	3.49	0.92	0.162
	CWG	24	3.32	0.45	
	RWG	25	3.64	0.49	

T30	NOG	10	3.22	0.65	0.108
	CWG	24	3.08	0.40	
	RWG	25	3.36	0.40	

T60	NOG	10	2.92	0.50	0.136
	CWG	24	2.88	0.34	
	RWG	25	3.09	0.34	

T120	NOG	10	2.73	0.33	0.445
	CWG	24	2.71	0.33	
	RWG	25	2.82	0.33	

T240	NOG	10	2.40	0.29	0.341
	CWG	24	2.47	0.29	
	RWG	25	2.56	0.32	

NOG, Nonobese group; CWG, corrected ideal body weight group; RWG, real body weight group; T0, T15, T30, T60, T120, and T240: baseline and 15, 30, 60, 120, and 240 min after magnesium sulfate administration. Two-wayrepeated-measures analysis of variance. The control group was not represented here because there were no changes in this group over time.

**Table 3:** Comparison of postoperative highest pain scores and morphine consumption during hospitalization.

Medians with the first and third quartiles of the highest postoperative pain scores and morphine consumption (mg·kg^−1^)						
Group	Median (pain)	25%	75%	Median (morphine)	25%	75%
CG	5.0	2	6	0.05	0.00	0.10
CWG	2.5	2	3	0.00	0.00	0.02
RWG	2.0	2	2	0.00	0.00	0.00
*p*	**0.006 ** ^*∗*^	N/A	N/A	**<0.001 ** ^*∗*^	N/A	N/A

CG, control group; CWG, corrected ideal body weight group; RWG, real body weight group, ^*∗*^Kruskal–Wallis. For highest pain, RWG *x* CG (*p*=0.005)-Bonferroni comparison, CWG *x* CG (*p*=0.016)-Bonferroni comparison, RWG *x* CWG (*p*=0.082)-Bonferroni comparison. For morphine consumption, RWG *x* CG (*p* ≤ 0.001)-Bonferroni comparison, CWG *x* CG (*p*=0.040)-Bonferroni comparison, RWG *x* CWG (*p*=0.323)-Bonferroni comparison.

**Table 4:** Pain scores after awakening.

Groups	30 min			60 min			120 min			240 min		
	Mean	Inf	Sup	Mean	Inf	Sup	Mean	Inf	Sup	Mean	Inf	Sup
CG	3.09	1.99	4.19	2.48	1.61	3.34	2.25	1.62	2.88	1.79	1.33	2.26
CWG	**1.05 ** ^*∗*^	0.58	1.51	1.67	1.10	2.23	1.79	1.54	2.04	1.96	1.67	2.25
RWG	**1.00 ** ^*∗∗*^	0.20	1.80	**1.25 ** ^*∗∗*^	0.85	1.65	1.60	1.39	1.81	1.88	1.63	2.13

Means and confidence intervals of pain scores (0, no pain; 10, worst imaginable pain) were recorded at four postoperative time points (in min). CG, control group; CWG, corrected ideal weight group; RWG, real weight group; Inf, lower limit; Sup, upper limit. One-way ANOVA. ^*∗*^*p* < 0.05 (Bonferroni comparisons): CWG < CG. ^*∗∗*^*p* < 0.05 (Bonferroni comparisons)–RWG < CG.

**Table 5:** Medians (minimum-maximum) and *p* value for comparison between groups.

	Latency (seconds)	Dur (seconds)
CWG (*n* = 24)	194.5 (148–276)	4262 (3405–5112)
RWG (*n* = 25)	196 (156–287)	4056 (2411–5530)
CG (*n* = 25)	204 (171–279)	3862 (3038–5005)
*p* value	0.651	0.181

CWG, Corrected ideal body weight; RWG, real body weight group; CG, control group; Dur, total duration.

## Data Availability

The datasets generated and analyzed in the present study are available from the corresponding author upon reasonable request.
